# Functional Identification Reveals That TaTGA16-2D Promotes Drought and Heat Tolerance

**DOI:** 10.3390/plants14142125

**Published:** 2025-07-09

**Authors:** Jingna Ru, Jiamin Hao, Xiaoqian Ji, Bingqing Hao, Jiale Yang, Hongtao Wang, Baoquan Quan, Pengyan Guo, Jiping Zhao, Chao Wang, Huawei Shi, Zhaoshi Xu

**Affiliations:** 1 The Industrial Crop Institute, Key Laboratory of Sustainable Dryland Agriculture of Shanxi Province, Shanxi Agricultural University, Taiyuan 030031, China; rujingna@sxau.edu.cn (J.R.); 2 Agricultural College, Shanxi Agricultural University, Jinzhong 030810, China; hjm4561999@163.com (J.H.); 3 State Key Laboratory of Crop Gene Resources and Breeding, Institute of Crop Sciences, Chinese Academy of Agricultural Sciences (CAAS), Beijing 100081, China; xuzhaoshi@caas.cn

**Keywords:** *TaTGA16-2D*, wheat, TGA transcription factor, expression profile, abiotic stress response

## Abstract

The TGACG motif-binding factor (TGA) family is an important group of basic region/leucine zipper (bZIP) transcription factors in plants, playing crucial roles in plant development and stress responses. This study conducted a comprehensive genome-wide analysis of the TGA transcription factor (TF) family in common wheat (*Triticum aestivum* L.). A total of 48 wheat *TGAs* were identified and classified into four subgroups. Collinearity analysis of the *TGAs* between wheat and other species identified multiple duplicated gene pairs and highlighted the presence of highly conserved *TGAs* in wheat. Whole-genome and segmental duplications were identified as the primary drivers of *TaTGA* expansion. Expression pattern analysis indicated that *TaTGAs* are involved in plant development and responses to abiotic stresses, including drought, heat, and cold treatment. Among these, *TaTGA16-2D* was significantly upregulated under both drought and heat stresses, showing more than a five-fold increase in expression. Subcellular localization confirmed its nucleus localization. Functional validation through ectopic expression in *Arabidopsis* demonstrated that transgenic lines overexpressing *TaTGA16-2D* exhibited significantly improved stress tolerance. Under heat stress, the survival rates of transgenic lines exceeded 34%, compared to less than 18% in wild-type plants. Overall, this study provides valuable insights into the evolution and functional roles of *TaTGAs* and identifies *TaTGA16-2D* as a promising candidate to enhance abiotic stress tolerance in wheat via molecular breeding.

## 1. Introduction

Transcription factors (TFs) play essential roles in regulating gene expression [1]. TGACG motif-binding factor (TGA) TFs belong to clade D of the basic region/leucine zipper (bZIP) family and specifically recognize and bind to the TGACG motif [2,3]. TGA TFs primarily function in regulating various physiological and metabolic processes in plants, as well as in responses to biotic and abiotic stresses [4]. Members of the TGA family interact with a variety of regulatory proteins, such as NPR1 (nonexpressor of pathogenesis-related genes 1), GRX480 (glutaredoxin 480), ERF72 (ethylene response factor 72), ARR2 (*Arabidopsis* response regulator 2), and SCL14 (scarecrow-like protein 14), and are involved in multiple stress and disease resistance signaling pathways, through which TGAs contribute to enhancing plant resilience to environmental challenges [5,6,7].

In *Arabidopsis*, the TGA TFs have been extensively studied and shown to participate in various biological processes. Several members are involved in plant development: *AtTGA1*, *AtTGA4*, and *AtTGA7* regulate flower development [8,9], while *AtTGA2*, *AtTGA5*, *AtTGA8*, and *AtTGA16* promote root growth by modulating redox homeostasis and oxidative stress responses [10]. In rice (*Oryza sativa*), *OsbZIP49*—a TGA-related bZIP TF—controls the tiller angle and plant architecture by influencing auxin homeostasis through the activation of indole-3-acetamide synthase [11].

TGA TFs also play key roles in biotic stress responses. For instance, *AtTGA9* and *AtTGA10* are involved in both pathogen defense and anther development [12]. Homologs of *Arabidopsis TGA* genes have been identified in other species. *BjCdR15* from *Brassica juncea*, a homolog of *AtTGA3*, is strongly induced by cadmium and other heavy metal stresses [13], while *AtTGA3* itself has been shown to participate in detoxification responses [14].

In addition to biotic stress, TGA factors are increasingly recognized for their roles in abiotic stress responses. *AtTGA1* enhances drought tolerance by promoting nitrate transport and uptake when overexpressed [15], and *AtTGA7* is involved in the drought stress response by regulating its target gene, *AtBG1* [16]. In soybean (*Glycine max*), the overexpression of *GmTGA17* enhances both drought and salt tolerance by modulating stress-responsive gene expression in the roots [17]. Similarly, *MhTGA2* from *Malus hupehensis* responds to low temperature, NaCl, and polyethylene glycol (PEG) treatment; the overexpression of *MhTGA2* improves salt and osmotic stress tolerance in transgenic *Malus hupehensis* and *Nicotiana tabacum* [18,19].

TGA TFs play a central role in wheat’s responses to abiotic and biotic stresses, as well as in growth and development. In wheat, *TaTGA2.1* was overexpressed in *Brachypodium distachyon*, and the transgenic plants showed enhanced resistance to *Fusarium graminearum* in both spikes and detached leaves [20]. Under drought and salinity stresses, as well as treatment with abscisic acid (ABA), *TaTGA2.2* expression is upregulated [21]. Additionally, the genes *TaTGA1a*, *TaTGA1b*, and *TaTGA4* have been successfully cloned using RT-PCR, and the expression pattern of *TaTGA2.2* has been preliminarily investigated using virus-induced gene silencing (VIGS) [22]. Research on *TGAs* in wheat remains relatively limited.

In this study, forty-eight *TaTGAs* were identified in wheat, followed by a comprehensive phylogenetic analysis. A systematic analysis was conducted on the gene structures and conserved domains of the *TaTGAs*. In addition, cis-acting regulatory elements and expression patterns were analyzed to explore their potential regulatory roles. The expression profiles of *TaTGAs* under drought, heat, and cold stress treatments were investigated to elucidate their involvement in environmental stress responses. Among them, a significantly differentially expressed gene, *TaTGA16-2D*, was selected for further functional analysis. Its role was ultimately validated through the phenotypic characterization of transgenic *Arabidopsis* plants. We hypothesize that *TaTGA16-2D* positively regulates abiotic stress tolerance and that its overexpression would enhance stress resilience in plants, making it a promising candidate for wheat molecular breeding.

## 2. Results

### 2.1. Identification of TaTGA Gene Family Members

**Figure 1:**
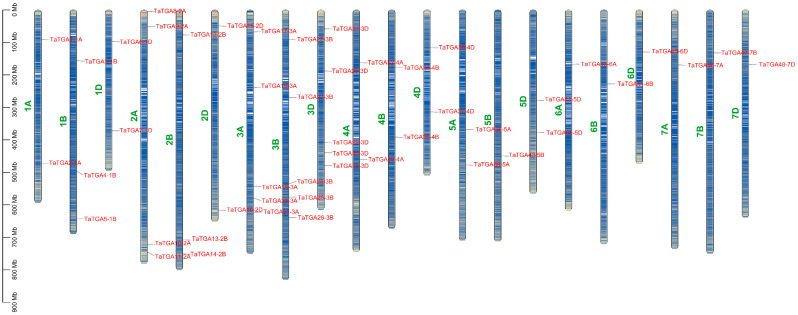
Chromosome localization of *TaTGA* gene family members in wheat. Left scale: megabases (Mb).

We conducted a comprehensive search of the wheat genome database using ten *Arabidopsis* and 16 riceTGA protein sequences as references. Based on the conserved domains and motifs, a total of 48 *TaTGAs* were identified through BLAST and HMMER analyses using TBtools-v2.119 (Figure 1; Table S1). The corresponding protein sequences ranged from 198 to 570 amino acids (aa) in length, with an average of 400 aa (Table S1). The molecular weight (kDa) values ranged from 21.66 kDa to 62.18 kDa, averaging 44.00 kDa. The isoelectric points (pI) ranged from 5.24 to 10.37, with 39.6% (19 out of 48) displaying alkaline characteristics. The instability index values ranged from 44.06 to 70.26, with all proteins exceeding 44, suggesting potential instability in vitro. The aliphatic index ranged from 66.70 to 96.86, with an average value of 78.68. The grand average of hydropathicity (GRAVY) values ranged from −0.74 to −0.10, all of which are negative, indicating that all TaTGA proteins are hydrophilic. Subcellular localization predictions showed that all TaTGA proteins were localized in the nucleus. To investigate their chromosomal distribution, the 48 *TaTGAs* were mapped onto the wheat genome. They were found to be unevenly distributed across all 21 chromosomes, with the number of *TaTGAs* per chromosome ranging from one to five (Figure S1).

### 2.2. Phylogenetic and Sequence Analyses of TaTGA Members

**Figure 2:**
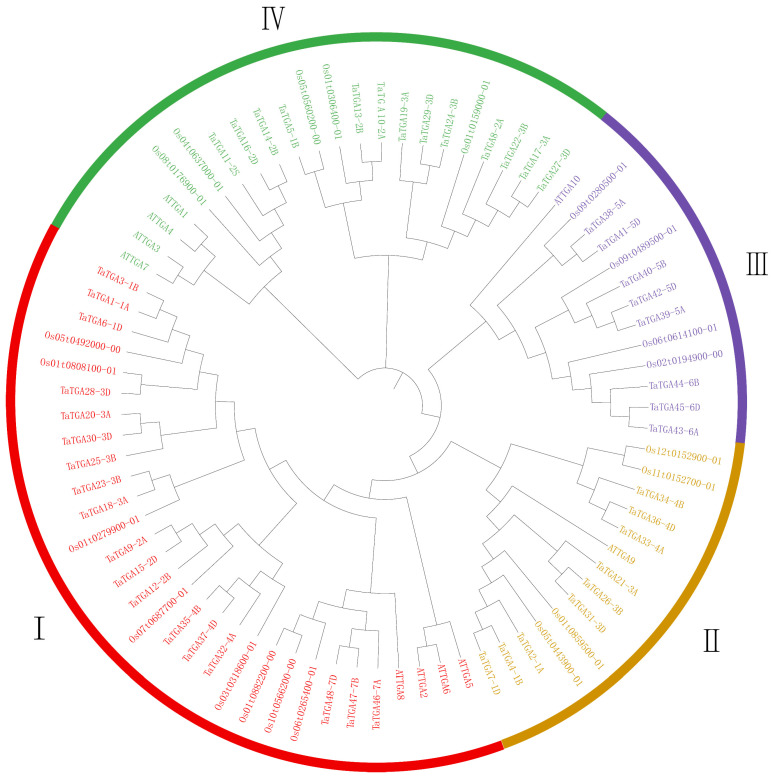
Phylogenetic analysis of *TaTGA* proteins in wheat, rice, and *Arabidopsis*. The TaTGA proteins were classified into four groups (I–IV), each marked with a different color. A total of 79 proteins were used to construct the neighbor-joining (NJ) phylogenetic tree by MEGA 11 with 1000 bootstrap replicates.

To understand the evolution of the TaTGA proteins, a phylogenetic analysis was conducted using a total of 79 TGA protein sequences, including 48 from *Triticum aestivum* (TaTGA), 10 from Arabidopsis, and 21 from rice (Figure 2; Table S2). The TGA proteins were classified into four groups (I–IV) based on their aa sequence similarity (Figure 2). Group I contained the largest number of TGA proteins across the three species. The analysis of the homoeologous relationships showed that the 48 TaTGAs corresponded to 48 homoeologs derived from 18 ancestral genes: 14 of these genes had all three homoeologs (one from each subgenome: A, B, and D), two had two homoeologs, and two had only a single homoeolog (Table S3).

### 2.3. Chromosomal Location and Duplication Events of TaTGA Genes

**Figure 3:**
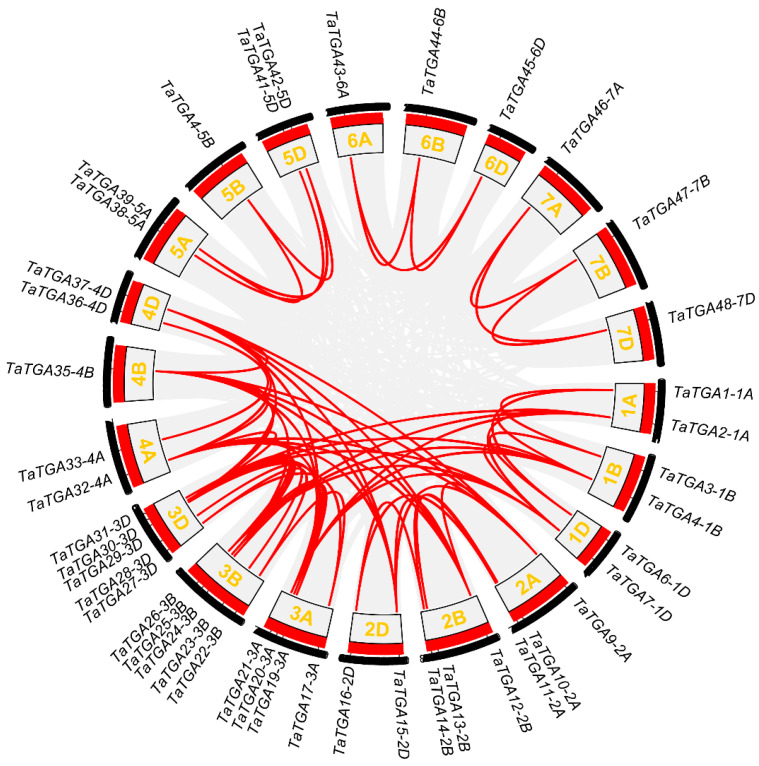
Wheat *TaTGA* gene family synteny analyses. Gene pairs formed due to segmental duplication are linked with red lines. The 21 wheat chromosomes (designated 1A to 7D) are numerically labeled within the circle.

**Figure 4:**
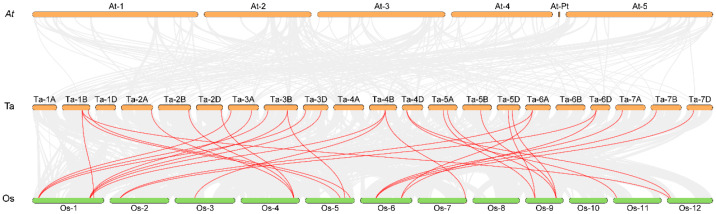
Collinearity analysis of *TaTGAs* between wheat (Ta), *Arabidopsis* (At), and rice (Os). The red lines represent interspecies collinear gene pairs.

Chromosomes 3A, 3B, and 3D together harbored nearly 31.3% of all *TaTGAs*. An analysis of the chromosomal translocations and inversions revealed that two triads—*TaTGA32-4A*/*TaTGA35-4B*/*TaTGA37-4D* and *TaTGA33-4A*/*TaTGA34-4B*/*TaTGA36-4D*—were involved in an inversion of chromosome 4A (Table S4). To elucidate the mechanisms underlying gene family expansion, we performed a collinearity analysis of the *TaTGAs* within the wheat genome. A total of 44 genes were located within syntenic blocks, forming 58 duplicated gene pairs (Figure 3; Table S5). Furthermore, all *TaTGAs* were found to originate from whole-genome duplication (WGD) or segmental duplication events (Table S4). To further explore the evolutionary history and homology of the *TGAs* across species, a comparative collinearity analysis was conducted between wheat, *Arabidopsis*, and rice. No collinear gene pairs were identified between wheat and *Arabidopsis*, while 31 collinear gene pairs were detected between wheat and rice (Figure 4), indicating a closer evolutionary relationship between wheat and rice.

The Ka/Ks ratios ranged from 0 (*TaTGA32-4A/TaTGA35-4B*, *TaTGA32-4A/TaTGA37-4D* and *TaTGA35-4B*/*TaTGA37-4D*) to 0.56 (*TaTGA23-3B/TaTGA28-3D*) (Figure S2, Table S5). These results suggest that most duplicated *TaTGA*s have undergone purifying selection.

### 2.4. Analysis of Gene Structures and Conserved Motifs of TaTGA Members

**Figure 5:**
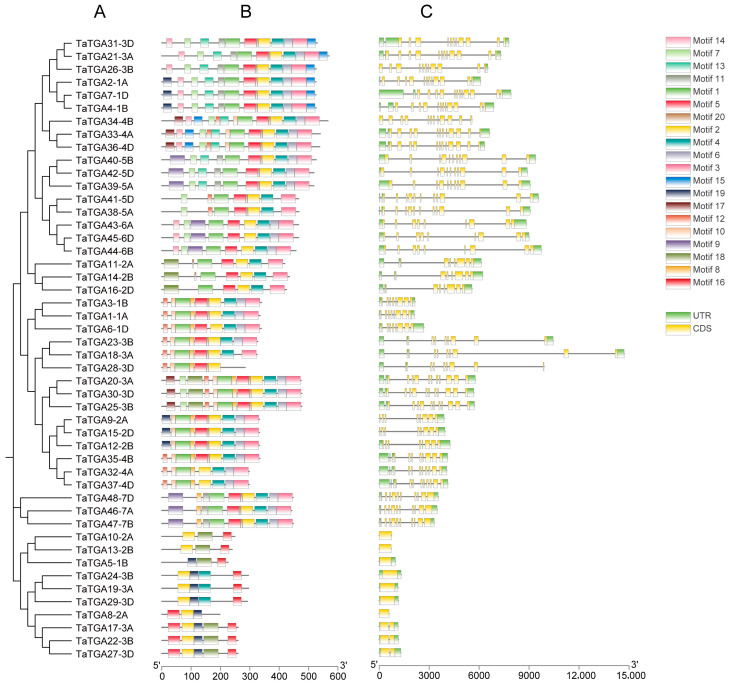
Phylogenetic relationships, motif distributions, and gene structures of 48 *TaTGA*s. (**A**) Multiple alignment of 48 TaTGA proteins. A total of 48 TaTGA proteins were used to construct the neighbor-joining (NJ) phylogenetic tree by MEGA 11 with 1000 bootstrap replicates. (**B**) Conserved motifs of 48 TaTGA proteins. (**C**) The exon–intron structure analysis of 48 *TaTGA*s.

The exon–intron structures and conserved motifs of the 48 *TaTGAs* were analyzed (Figure 5). Among the 48 genes, seven lacked introns, while the remaining *TaTGAs* contained between one and 11 introns (Figure 5A,B; Table S6). *TaTGA2-1A*, *TaTGA4-1B*, *TaTGA7-1D*, *TaTGA21-3A*, *TaTGA33-4A*, *TaTGA34-4B*, and *TaTGA36-4D* had the highest numbers of exons and introns (12 and 11, respectively). In terms of UTRs, seven genes lacked a 5′ UTR and five genes lacked a 3′ UTR. *TaTGA32-4A*, *TaTGA35-4B*, and *TaTGA37-4D* possessed the longest 5′ and 3′ UTRs, with three and one regions, respectively. In Arabidopsis, most TGA genes, including AtTGA1 through AtTGA6, shared a uniform structure of eight exons and seven introns. AtTGA9 had the most complex structure among them. Regarding UTRs, AtTGA2 and AtTGA7 had the highest numbers of 5′ UTRs (three each), while the other TGA genes had simpler UTR arrangements. In rice, the gene structures were similar to those of Arabidopsis in several TGA members. Os01t0279900, Os06t0265400, Os03t0318600, Os05t0492000, and Os07t0687700 all had eight exons and seven introns. However, Os01t0859500, Os12t0152900, and Os11t0152700 exhibited the most complex structures, with 12 exons and 11 introns—paralleling the complexity seen in certain wheat TaTGA genes. In terms of UTRs, Os06t0265400, Os07t0687700, and Os04t0637000 showed the most extended 5′ UTRs, each with three regions (Table S6).

Genes grouped in the same phylogenetic branch, typically homologous, generally exhibited similar gene structures. However, structural divergence was observed among some homologous genes; for example, *TaTGA21-3A*, *TaTGA26-3B*, and *TaTGA31-3D* differed in their exon, intron, and UTR compositions. A total of 20 conserved motifs were seen, each ranging from six to 50 aa in length (Figure 5C; Table S7). Among these, motif 1 contained highly conserved sequence domains corresponding to PF14144 and PF00170, indicating potential functional importance.

### 2.5. Analysis of Promoter Cis-Elements of TaTGA Members

In the promoter regions of the 48 *TaTGAs*, we identified a total of 528 potential cis-elements (Table S8). These elements were grouped into four primary categories: plant growth-related elements (12%), hormone-responsive elements (26%), environmental stress-responsive elements (34%), and light-responsive elements (28%) (Figure 3). Notably, the environmental stress-responsive category included 177 drought-responsive elements, while the hormone-responsive category contained 111 methyl jasmonate (MeJA)-responsive elements. These findings suggest that *TaTGAs* may play significant roles in abiotic stress responses, particularly in drought stress adaptation. Furthermore, meristem expression elements were the most abundant, totaling 63, suggesting a potential role for *TaTGAs* in regulating plant growth and development (Table S8).

### 2.6. Expression Pattern Analysis of TaTGA Genes

**Figure 6:**
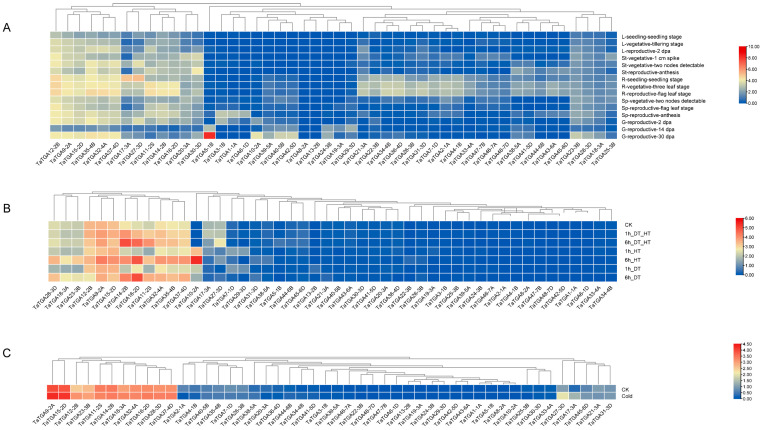
Heatmaps show the expression profiles of 48 *TaTGA* genes across (**A**) different wheat tissue types and developmental stages, (**B**) drought and high-temperature treatments, and (**C**) cold treatment. Expression values are presented as TPM and were normalized for clustering analysis.

An analysis of the *TaTGA* expression patterns across various tissue types and developmental stages revealed that *TaTGA8-2A* and *TaTGA13-2B* exhibited extremely low transcription levels in the examined samples (Figure 6A; Table S9). Multiple genes exhibited distinct tissue-specific expression profiles. For example, *TaTGA5-1B* was highly expressed in the grain, while *TaTGA4-1B*, *TaTGA7-1D*, *TaTGA22-3B*, and *TaTGA34-4B* formed a distinct cluster with strong expression in the roots. In contrast, some genes were constitutively expressed across various tissue types. Notably, *TaTGA12-2B*, *TaTGA9-2A*, *TaTGA15-2D*, *TaTGA32-4A*, and *TaTGA37-4D* showed high transcription levels throughout plant development (Figure 6A). Overall, the *TaTGAs* exhibited pronounced tissue-specific expression, suggesting potential roles in diverse physiological processes and developmental regulation.

In the analysis of the *TaTGAs*’ expression patterns under abiotic stress conditions, thirteen genes were differentially expressed under the two stress conditions, including *TaTGA9-2A*, *TaTGA11-2A*, *TaTGA12-2B*, *TaTGA14-2B*, *TaTGA15-2D*, *TaTGA16-2D*, *TaTGA17-3A*, *TaTGA18-3A*, *TaTGA23-3B*, *TaTGA28-3D*, *TaTGA32-4A*, *TaTGA35-4B*, and *TaTGA37-4D* (Figure 6B; Table S9). Additionally, 10 genes were differentially expressed under cold treatment, namely *TaTGA9-2A*, *TaTGA12-2B*, *TaTGA15-2D*, *TaTGA16-2D*, *TaTGA17-3A*, *TaTGA18-3A*, *TaTGA23-3B*, *TaTGA27-3D*, *TaTGA28-3D*, and *TaTGA37-4D, TaTGA27-3D.* Notably, *TaTGA27-3D* exhibited the strongest response to cold stress among all genes analyzed (Figure 6C). Based on their responsiveness to abiotic stresses and their consistently high expression across developmental stages, six genes were selected for further analysis: *TaTGA10-2A*, *TaTGA11-2A*, *TaTGA14-2B*, *TaTGA16-2D*, *TaTGA17-3A*, and *TaTGA27-3D*.

### 2.7. TaTGAs Are Involved in Abiotic Stress Responses

**Figure 7:**
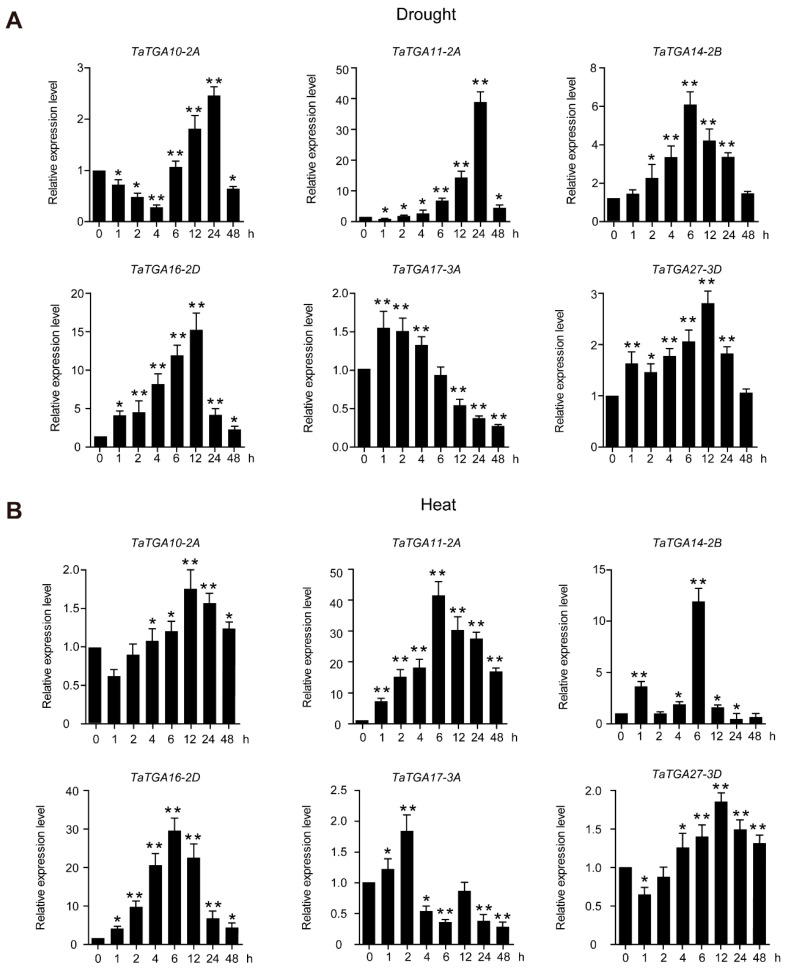
qRT-PCR analysis of 6 *TaTGAs* under drought (**A**) and heat (**B**) treatments. Seven-day-old wheat seedlings were used for drought (10% PEG) and heat (42 °C) treatments. The relative expression levels of target genes were normalized to the expression of *TaACTIN.* The data represent the mean ± SD of three biological replications. An ANOVA test was used to analyze significant differences (* *p* < 0.05, ** *p* < 0.01).

The expression patterns of six *TaTGAs* were examined using qRT-PCR (Figure 7). Under drought treatment, *TaTGA11-2A*, *TaTGA14-2B*, and *TaTGA16-2D* were significantly upregulated (>five-fold), reaching their respective expression peaks at 24 h, 6 h, and 24 h (Figure 7A), indicating temporal differences in stress responsiveness. In contrast, the other three genes exhibited only slight responses to dehydration (Figure 7A). Following heat treatment, a similar expression trend was observed: *TaTGA11-2A*, *TaTGA14-2B*, and *TaTGA16-2D* were again significantly upregulated (>five-fold), all peaking at 6 h (Figure 7B), suggesting a more rapid transcriptional response to heat stress compared to drought. These results imply that, while the same genes respond to multiple stress conditions, their expression dynamics—such as the peak time and intensity—differ according to the type of abiotic stimulus. These findings demonstrate that, although some *TaTGAs* are repeatedly induced under various stresses, their differential expression patterns—both in magnitude and timing—reflect nuanced regulatory roles in distinct stress signaling pathways. Among them, *TaTGA16-2D* was selected for further functional analysis due to its robust and consistent response.

### 2.8. TaTGA16-2D Was Located in the Nucleus

**Figure 8:**
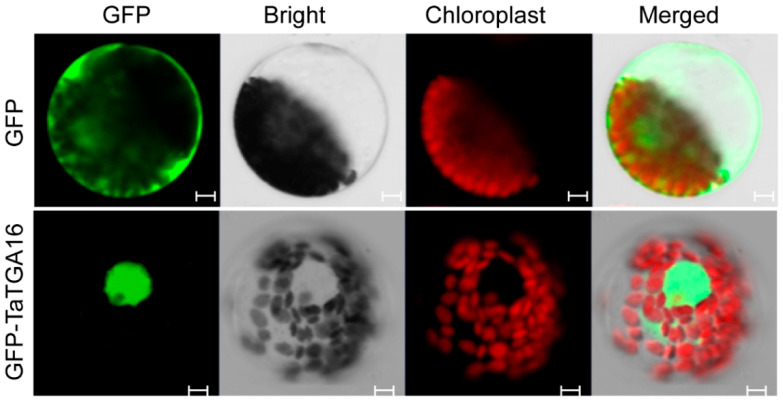
Subcellular localization of TaTGA16-2D-GFP fusion protein in wheat protoplasts. Scale bars = 5 μm. The results are representative of three independent experiments. The p16318hGFP control vector and recombinant constructs were transiently expressed in wheat protoplasts. Green indicates GFP signals, and red indicates chloroplast autofluorescence. A negative control was performed using an empty GFP vector (without the target gene), to exclude non-specific localization caused by the GFP protein itself. Results were observed after transformation for 18 h with confocal microscopy.

The subcellular localization of the 48 TaTGAs was predicted using BUSCA (Table S1). To experimentally validate the localization of TaTGA16-2D, transient expression assays were conducted. TaTGA16-2D was predominantly localized to the nucleus (Figure 8).

### 2.9. TaTGA16-2D Enhances Drought Tolerance in Arabidopsis

**Figure 9:**
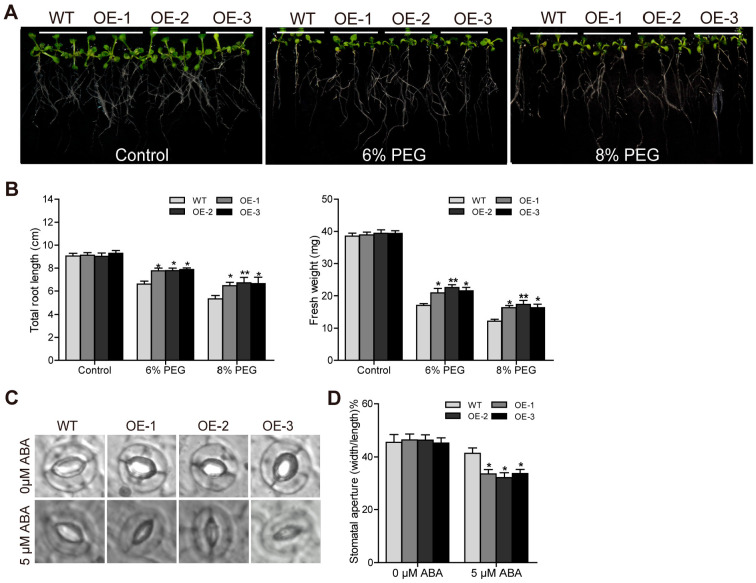
Phenotypic analysis of *TaTGA16-2D* transgenic plants under drought treatment: (**A**) root length and (**B**) total root length and fresh weight of WT and transgenic lines; (**C**) stomatal phenotype; (**D**) pore opening. Values represent means ± SD from three biological replicates. Asterisks indicate significant differences compared to WT (* *p* < 0.05; ** *p* < 0.01).

The total root length and fresh weight were measured in root growth assays. Under control conditions, there were no significant differences in the root length or fresh weight between transgenic and wild-type (WT) plants. However, under 6% PEG treatment, although growth was inhibited in both genotypes, the growth inhibition was significantly reduced in the transgenic lines relative to the WT plants, as evidenced by longer roots and higher fresh weights. Under 8% PEG treatment, both WT and transgenic plants exhibited more severe growth inhibition; however, the transgenic lines maintained significantly greater root lengths and fresh weights compared to WT plants, demonstrating enhanced tolerance (Figure 9A,B).

To further investigate whether *TaTGA16-2D* influenced stomatal behavior under ABA treatment, we performed a stomatal aperture assay (Figure 9C,D). Leaves from the same position in the WT and *TaTGA16-2D*-overexpressing *Arabidopsis* were excised and incubated under strong light for 3 h in a stomatal opening buffer. The leaves were then transferred to a buffer containing either 0 μM or 5 μM ABA and incubated for an additional 2 h. In the absence of exogenous ABA, there was no significant difference in stomatal opening between the WT and *TaTGA16-2D*-overexpressing plants. However, after treatment with 5 μM ABA, the stomatal apertures decreased in both lines, but the reduction was significantly greater in the *TaTGA16-2*-overexpressing plants compared to the wild type. These results suggest that *TaTGA16-2* enhances the sensitivity of *Arabidopsis* to ABA and plays a role in regulating ABA-mediated stomatal closure (Figure 9C,D).

### 2.10. TaTGA16-2D Increases Heat Tolerance in Arabidopsis

**Figure 10:**
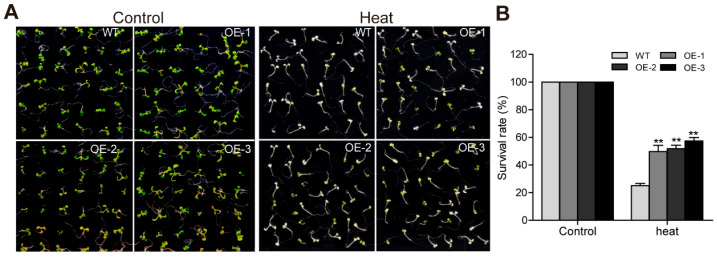
Phenotype analysis of *TaTGA16-2D* under heat stress. Phenotypes (**A**) and survival rates (**B**) of WT and *TaTGA16-2D* transgenic lines under heat stress. Seven-day-old seedlings were placed at 42 °C. Values are means ± SD of three replicates. Significant differences were assessed using ANOVA (** *p* < 0.01).

*TaTGA16-2D* was upregulated over 30-fold under heat stress. Transgenic *Arabidopsis* lines overexpressing *TaTGA16-2D* exhibited significantly higher survival rates under heat stress compared to WT (>34% vs. <18%), suggesting that the ectopic expression of *TaTGA16-2D* enhances heat tolerance (Figure 10).

## 3. Discussion

TGA TFs mediate various processes, including plant defense and growth, and have been proven to play crucial roles in regulating plant stress responses, as well as growth and development [23]. To date, the genomic identification of TGA TFs has primarily been conducted in *Arabidopsis* [24], rose (*Rosa* spp.) [25], common bean (*Phaseolus vulgaris* L.) [26], soybean [27], etc. The numbers of identified TGA TFs vary across species, from six to 40 [4]. However, few studies have been conducted on wheat, and the identification of *TGAs* remains particularly limited. Here, this study performed a comprehensive analysis of the 48 TaTGA TFs in wheat.

TGA TFs are more numerous in wheat than in *Arabidopsis* and rice [23]. The 48 TaTGA TFs are unevenly distributed across chromosomes (Figure 1); for instance, chromosome 1 harbors the most in rice, and chromosome 5 contains the most TGAs in *Arabidopsis* [28]. In wheat, chromosome 3 contains the highest number of *TaTGAs* (Figure 1). While gene family expansion is generally driven by genome polyploidy and gene duplication, an analysis of the wheat *TGA* gene family revealed 18 segmentally duplicated gene pairs but no tandem duplication events (Table S3). All wheat *TaTGAs* were determined to have originated from WGD/segmental duplication events, indicating that WGD/segmental duplication is the primary force driving expansion of *TGAs* in wheat (Figure 3). A phylogenetic analysis was conducted comparing *TGAs* from wheat, *Arabidopsis*, and rice (Figure 2). The results indicated that TGA sequences and functions are conserved across these species. However, wheat TGAs show a closer phylogenetic relationship and higher collinearity with rice TGAs than with those of *Arabidopsis* (Figure 4). This evolutionary divergence may help to explain why the TGAs are classified into five subgroups in *Arabidopsis* but only four in wheat. The 10 *AtTGAs* in *Arabidopsis* are divided into five groups, with *AtTGA9* and *AtTGA10* assigned to the same group [4]. However, in wheat, these two *TGAs* are separated into distinct groups due to the higher number of homologous genes corresponding to each (Figure 2).

A total of 20 conserved motifs were identified across the TaTGA proteins. Notably, motif 1 contained highly conserved sequences corresponding to the bZIP TF domain (PF00170) and the seed dormancy control domain (PF14144). As expected, proteins within the same phylogenetic branch typically shared similar motif compositions and arrangements (Figure 5). Nevertheless, some proteins in the same clade displayed variations in motif distribution, which may reflect differences in exon–intron organization and potential functional divergence.

Cis-elements in promoter regions play a critical role in regulating gene expression. Several stress-responsive cis-elements were identified in the promoter regions of *TaTGAs* that exhibited differential responses to abiotic stress (Figure 5), suggesting their potential involvement in stress adaptation mechanisms. These findings lay a foundation for the functional characterization of *TaTGAs* in wheat. Increasing evidence indicates that TaTGA TFs play important roles in plant responses to abiotic stresses. However, only a few stress-related TGA genes have been functionally characterized in wheat to date, such as *TaTGA2.1, TaTGA2.2* [29], and *TaTGA1* [22], etc.

The functional analysis of the *TaTGAs* expressed in different organs has provided novel insights into their regulatory roles in plant growth and organ-specific responses to drought, heat, and cold (Figure 6 and Figure 7). Through expression analysis, we selected six potential candidate genes that responded to various stresses. Among them, *TaTGA27-3D* showed no significant response to drought and heat stress but responded significantly to cold stress (Figure 6 and Figure 7); *TaTGA11-2A* and *TaTGA16-2D* exhibited the most significant responses to drought and heat stress. These genes will be the focus of our next study, and elucidating their response mechanisms will contribute to uncovering the complex pathways through which *TGAs* mediate responses to various stresses.

The transcriptomic analysis revealed that *TaTGA16-2D* was significantly upregulated in response to multiple abiotic stresses, prompting its selection for further functional analysis. Under drought and heat stress conditions, *TaTGA16-2D* transgenic *Arabidopsis* plants exhibited significantly greater root lengths and fresh weights or higher survival rates compared to controls (Figure 9 and Figure 10). Furthermore, under drought conditions, *TaTGA16-2D* enhances the sensitivity of *Arabidopsis* to ABA and plays a key role in regulating ABA-mediated stomatal closure (Figure 9). These results suggest that *TaTGA16-2D* plays a positive regulatory role in drought and heat stress responses. *Arabidopsis TGA4*, a homolog of *TaTGA16-2D*, is highly expressed in the roots and plays a crucial role in salicylic acid-mediated immune responses [30]. Additionally, *TGA4* functions as a key TF in nitrate-mediated nitrogen signaling [31]. These findings highlight the important role of *TGA4* in both biotic and abiotic stress responses, providing a valuable reference for further research on *TaTGA16-2D*. Given its strong stress-responsive expression and positive effects in *Arabidopsis*, *TaTGA16-2D* represents a promising candidate gene to enhance abiotic stress resistance in wheat. In summary, this study provides the first comprehensive genome-wide identification and characterization of the TGA transcription factor family in wheat. This study not only deepens our understanding of the evolutionary and functional diversity of *TGAs* in wheat but also identifies *TaTGA16-2D* as a promising gene to improve abiotic stress resistance in crops, thereby offering valuable resources for molecular breeding.

## 4. Materials and Methods

### 4.1. Identification of TGA Genes in Wheat

First, wheat reference sequences were downloaded from Ensembl Plants (http://plants.ensembl.org/index.html (accessed on 13 March 2025)). The TGA sequences of *Arabidopsis* and *Oryza sativa* L. were obtained from the TAIR (https://www.arabidopsis.org/ (accessed on 10 March 2025)) and RGAP databases (http://rice.uga.edu/ (accessed on 10 March 2025)), respectively. These sequences were then used as queries in BLASTP searches against the wheat protein database, with an *E*-value threshold of <1 × 10^−10^. Additionally, the Hidden Markov Model (HMM) profiles for the bZIP TF (PF00170) and seed dormancy control (PF14144) were downloaded from the Pfam database for the identification of TGA proteins (http://pfam.xfam.org/ (accessed on 8 April 2025)). We conducted an HMM search to build a wheat-specific HMM profile using the Simple HMM Search tool in TBtools v2.119 (https://github.com/CJ-Chen/TBtools (accessed on 15 April 2025)) [32], with an *E*-value threshold of <1 × 10^−10^. The results obtained from both BLAST and HMM analyses were integrated. Following validation, all putative *TaTGAs* were successfully identified (Table S1). Subcellular localizations were predicted through the Plant-mPLoc website (http://www.csbio.sjtu.edu.cn/bioinf/plant-multi/ (accessed on 26 April 2025)).

### 4.2. Phylogenetic Analysis of the TaTGA Gene Family

Multiple alignment of the conserved TaTGA protein sequences was conducted using Clustal X, and the optimal substitution model was applied to construct an interspecific phylogenetic tree using MEGA 11 [33]. The evolutionary tree was constructed using the neighbor-joining (NJ) method, with 1000 bootstrap replicates.

### 4.3. Analysis of Conserved Motifs and Cis-Acting Elements in Wheat TaTGA Genes

The MEME program (Multiple EM for Motif Elicitation; http://meme-suite.org/tools/meme (accessed on 27 April 2025)) was applied to analyze motifs; we set the motif number to 20 and downloaded the MAST XML output files from the analysis results. PlantCARE was used to analyze the cis-acting elements in the 2000-base-pair (bp) promoter region upstream of the ATG start codons of the *TaTGAs* (http://bioinformatics.psb.ugent.be/webtools/plantcare/html/ (accessed on 27 April 2025)) [34]. The Batch CD-Search tool on NCBI was used to analyze conserved domains. The results were subsequently visualized using the Gene Structure View (Advanced) module in TBtools v2.119 [32].

### 4.4. Chromosome Localization, Gene Duplication, and Collinearity Analysis of TaTGA Gene Family

Segmental and tandem duplication events within the *TaTGAs* were identified using the integrated MCScanX module in TBtools v2.119 [34]. The nonsynonymous substitution rate (Ka), synonymous substitution rate (Ks), and Ka/Ks ratios were subsequently calculated using the same software. In addition, homology relationships among *TGAs* from *Arabidopsis*, rice, and wheat were analyzed and visualized to explore evolutionary conservation across species.

### 4.5. Transcriptome Analysis of TaTGA Gene Family in Different Tissue Types and Under Abiotic Stress

Transcriptomic data from the Wheat Expression Browser (http://www.wheat-expression.com (accessed on 11 April 2025)) were used (choulet_URGI) [35,36]. Datasets SRP045409 and SRP043554 were obtained to investigate the expression levels of *TaTGAs* under drought, heat, and cold abiotic stresses [37,38]. Heatmaps were generated using the HeatMap tool in TBtools v2.119 [34].

### 4.6. TaTGA Expression Profiling and Real-Time PCR (qRT-PCR)

The wheat cultivar Xiaobaimai was used to analyze gene expression patterns. The plants were cultured under conditions of 16 h of light and 8 h of darkness, with temperatures of 22 °C and 60% humidity. Seven-day-old wheat seedlings were subjected to drought stress (10% PEG 200) and heat stress (42 °C). Samples were systematically collected at 0, 1, 2, 4, 6, 12, 24, and 48 h after treatment. The leaf samples were processed to extract RNA using the RNA plant extraction kit (TIANGEN, Beijing, China). cDNA was synthesized using the ReverTra Ace qPCR RT Master Mix (Toyobo, Kyoto, Japan). Six candidate genes, namely *TaTGA10*, *TaTGA11*, *TaTGA14*, *TaTGA16*, *TaTGA17*, and *TaTGA27*, were subjected to qRT-PCR analysis using Super Real PreMix Plus (SYBR Green) (TIANGEN, China). The thermal cycling conditions were as follows: denaturation at 95 °C for 2 min, followed by 45 cycles at 94 °C for 20 s, 60 °C for 15 s, and 72 °C for 20 s. The expression of genes was normalized to that of a housekeeping gene (*TaACTIN*, accession no. AB181991) [39]. Each experiment was performed with three biological replicates. Relative expression levels were calculated using the 2^−ΔΔCT^ method [40]. All primers are listed in Table S10.

### 4.7. Subcellular Localization

The coding sequence (CDS) of *TaTGA16-2D* was inserted into the p16318hGFP vector under control of the CaMV35S promoter. The p16318hGFP-TaTGA16-2D and control GFP plasmids were transformed into wheat protoplasts mediated by PEG4000. Wheat protoplasts were isolated from five-day-old seedlings [41]. The fluorescence signals were captured by a confocal laser scanning microscope (LSM700; Carl Zeiss, Jena, Germany). Primers used for amplification are shown in Table S10.

### 4.8. Generation of Transgenic Arabidopsis

The *Arabidopsis* cultivar Columbia-0 was used for transformation. The CDS excluding the termination codons of TaTGA16-2D was cloned into plant expression vector pCAMBIA2300 under the control of the CaMV35S promoter. After sequencing, the correct plasmid pCAMBIA2300-TaTGA16-2D was transformed into *Arabidopsis* by the Agrobacterium tumefaciens-mediated floral dip method [42]. The transformed seeds were surface-sterilized with sodium hypochlorite and selected on 1/2 MS medium containing 50 μg/mL Kanamycin and then transferred to soil (rich soil–vermiculite = 1:1). T_3_ generation plants were grown at 22 °C with 60% relative humidity under a 16 h light/8 h dark photoperiod. Three homozygous T_3_ lines were selected for the following phenotypic analysis.

### 4.9. Abiotic Stress Treatments of Transgenic Arabidopsis

For drought stress treatment, five-day-old transformed seedlings were transferred to MS medium supplemented with 6% or 8% PEG, or to PEG-free medium as a control, and grown for an additional 7 days. The total root lengths and fresh weights were then measured.

To investigate the role of *TaTGA16-2D* in ABA-mediated stomatal regulation, a stomatal aperture assay was performed. The stomatal opening solution was prepared with 10 mM MES-Tris (pH 6.15), 10 mM KCl, and 7.5 mM iminodiacetic acid. Mature leaves from the same position in wild-type and *TaTGA16-2D*-overexpressing *Arabidopsis* were excised and floated on the stomatal opening solution under strong light for 3 h to induce stomatal opening. Subsequently, the leaves were transferred to stomatal opening solutions containing 0 μM, 5 μM, or 10 μM ABA and incubated under the same light conditions for an additional 2 h. Stomatal apertures were observed under a light microscope, and images were captured. Three biological replicates were performed for each treatment, with at least 30 stomata measured per replicate. The stomatal pore length and width were measured using the ImageJ software version 1.54p [43], and stomatal opening was calculated as

Stomatal opening (%) = (width/length) × 100.

For heat stress treatment, the transformed seeds were grown on MS medium for 5 days and then exposed to 42 °C for 1 day, followed by transfer to 4 °C for 14 h. Plants were subsequently returned to normal conditions at 22 °C for 3 days, after which survival rates were recorded. The experiment was performed in triplicate.

## 5. Conclusions

This study comprised a comprehensive study of the 48 members of the wheat *TGA* gene family, which were categorized into four groups. These genes were then subjected to comprehensive analyses, including phylogeny, chromosomal distribution, duplication events, gene structure, and protein motif analyses, transcriptome profiling, and cis-acting element identification. Subsequently, the expression levels of six candidate genes were validated by qRT-PCR. As a result, these *TaTGAs* underwent several segmental duplication events, which played a dominant role in the expansion of the *TaTGA* family. In particular, *TaTGA16-2D* was further investigated and found to be involved in tolerance to drought and heat stress. These results lay a foundation for future studies to characterize wheat TGA genes.

## Data Availability

Data are contained within this article and Supplementary Materials.

## Supplementary Materials

The following supporting information can be downloaded at: https://www.mdpi.com/article/10.3390/plants14142125/s1, Figure S1. The distribution frequency of *TaTGA* genes per chromosome. Figure S2. Histogram of distribution frequency of pairwise Ka/Ks ratios for duplicated *TaTGAs*. Ka, nonsynonymous substitution rate; Ks, synonymous substitution rate. Figure S3. Cis-element distribution of 48 *TaTGA* genes in different biological processes. Table S1. The detailed information of 48 wheat *TaTGA* genes. Table S2. List of sequence IDs used in the phylogenetic analysis. Table S3. Distribution pattern of *TaTGA* homeologs. Table S4. Chromosome inversion and translocation events in *TaTGA* homeologs. Table S5. Ka/Ks values of duplicated *TaTGA* genes. Table S6. Exon and intron numbers of 48 *TaTGA* genes. Table S7. List of conserved motifs in TaTGA proteins. Table S8. Cis-elements in the promoter regions of 48 *TaTGA* genes. Table S9. The TPM values of 48 *TaTGA* genes from transcriptome data. Table S10. The primers used in this study.

## References

[1] Kajla M., Roy A., Singh I.K., Singh A. (2023). Regulation of the regulators: Transcription factors controlling biosynthesis of plant secondary metabolites during biotic stresses and their regulation by miRNAs. Front. Plant Sci..

[2] Katagiri F., Lam E., Chua N.H. (1989). Two tobacco DNA-binding proteins with homology to the nuclear factor CREB. Nature.

[3] Johnson C., Boden E., Arias J. (2003). Salicylic acid and npr1 induce the recruitment of *trans*-activating tga factors to a defense gene promoter in *arabidopsis*. Plant Cell.

[4] Lu C., Liu X., Tang Y., Zhang Y., Yang J., Li L., Zhu P., Dong Z., Pan D. (2024). A comprehensive review of TGA transcription factors in plant growth, stress responses, and beyond. J. Integr. Plant. Biol..

[5] Lam E., Benfey P.N., Gilmartin P.M., Fang R.X., Chua N.H. (1989). Site-specific mutations alter in vitro factor binding and change promoter expression pattern in transgenic plants. Proc. Natl. Acad. Sci. USA.

[6] Buttner M., Singh K.B. (1997). *Arabidopsis thaliana* ethylene-responsive element binding protein (AtEBP), an ethylene-inducible, GCC box DNA-binding protein interacts with an ocs element bindingprotein. Proc. Natl. Acad. Sci. USA.

[7] Choi J., Huh S.U., Kojima M., Sakakibara H., Paek K.H., Hwang I. (2010). The cytokinin-activated transcription factor arr2 promotes plant immunity via tga3/npr1-dependent salicylic acid signaling in *arabidopsis*. Dev. Cell..

[8] Wang Y., Salasini B.C., Khan M., Devi B., Bush M., Subramaniam R., Hepworth S. (2019). Clade i tgacg-motif binding basic leucine zipper transcription factors mediate blade-on-petiole-dependent regulation of development. Plant Physiol..

[9] Xu X., Xu J., Yuan C., Hu Y., Qin C. (2021). Characterization of genes associated with TGA7 during the floral transition. BMC Plant Biol..

[10] Hu X., Yang L., Ren M., Liu L., Fu J., Cui H. (2022). TGA factors promote plant root growth by modulating redox homeostasis or response. J. Integr. Plant Biol..

[11] Ding C., Lin X., Zuo Y., Yu Z., Baerson S.R., Pan Z., Zeng R., Song Y. (2021). Transcription factor OsbZIP49 controls tiller angle and plant architecture through the induction of indole-3-acetic acid-amido synthetases in rice. Plant J..

[12] Murmu J., Bush M.J., DeLong C., Li S., Xu M., Khan M., Malcolmson C., Fobert P.R., Zachgo S., Hepworth S.R. (2010). *Arabidopsis* basic leucine-zipper transcription factors TGA9 and TGA10 interact with floral glutaredoxins ROXY1 and ROXY2 and are redundantly required for anther development. Plant Physiol..

[13] Georgieva T., Christov N.K., Djilianov D. (2012). Identification of desiccation-regulated genes by cDNA-AFLP in Haberlea rhodopensis: A resurrection plant. Acta Physiol. Plant..

[14] Fang H., Liu Z., Long Y., Liang Y., Pei Y. (2017). The Ca/calmodulin2-binding transcription factor TGA3 elevates LCD expression and H S production to bolster Cr tolerance in *Arabidopsis*. Plant J..

[15] Zhong L., Chen D., Min D., Li W., Xu Z., Zhou Y., Li L., Chen M., Ma Y. (2015). AtTGA4, a bZIP transcription factor, confers drought resistance by enhancing nitrate transport and assimilation in *arabidopsis thaliana*. Biochem. Biophys. Res. Commun..

[16] Lee K.H., Piao H.L., Kim H.Y., Choi S.M., Jiang F., Hartung W., Hwang I., Kwak J.M., Lee I.J., Hwang I. (2006). Activation of glucosidase via stress-induced polymerization rapidly increases active pools of abscisic acid. Cell.

[17] Li B., Liu Y., Cui X.Y., Fu J.D., Zhou Y.B., Zheng W.J., Lan J.H., Jin L.G., Chen M., Ma Y.Z. (2019). Genome-Wide characterization and expression analysis of soybean TGA transcription factors identified a novel TGA gene involved in drought and salt tolerance. Front. Plant Sci..

[18] Stracke R., Favory J.-J., Gruber H., Bartelniewoehner L., Bartels S., Binkert M., Funk M., Weisshaar B., Ulm R. (2010). The *arabidopsis* bZIP transcription factor HY5 regulates expression of thePFG1/MYB12gene in response to light and ultraviolet-B radiation. Plant Cell Environ..

[19] Du X., Du B., Chen X., Zhang S., Zhang Z., Qu S. (2014). Overexpression of the *MhTGA2* gene from crab apple (*Malus hupehensis*) confers increased tolerance to salt stress in transgenic apple (*Malus domestica*). J. Agric. Sci..

[20] Su P., Guo X., Fan Y., Wang L., Yu G., Ge W., Zhao L., Ma X., Wu J., Li A. (2018). Application of Brachypodium genotypes to the analysis of type II resistance to Fusarium head blight (FHB). Plant Sci..

[21] Guo S., Ren H., Zhang Y., Feng C., Feng H., Wang X., Kang Z., Zhang X. (2020). Characterization and functional analyses of wheat disease resistance-related gene *TaTGA2.2* in the interaction between wheat and stripe rust. J. Triticeae Crops..

[22] Xu Z., Zhang H., Mo Q., Lv S., Ji W. (2018). Expression analysis of wheat transcription factor TaTGA1 gene responding to infection of powdery mildew. Acta Phytopathol. Sin..

[23] Xu D., Lin D., Li S., Adan L., Han Y., Zhang Y., Liu T., Qi H. (2024). Research progress on the role of TGA transcription factor in regulating plant stress response and growth and development. Plant Physiol. J..

[24] Christiane G. (2013). From pioneers to team players: TGA transcription factors provide a molecular link between different stress pathways. Mol. Plant-Microbe Interact. MPMI.

[25] Gao P., Zhang H., Yan H., Chen Y., Fan Y., Yan B., Qiu X. (2021). Analysis of gene structure of *TGAs* and function of *RcTGA2* in *Rosa* chinensis Jacq. Old Blush against Botrytis cinerea. Plant Physiol. J..

[26] Liu Y., Huang Y., Li Z., Feng M., Ge W., Zhong C., Xue R. (2023). Genome-wide identification of the TGA genes in common bean (*Phaseolus vulgaris*) and revealing their functions in response to *fusarium oxysporum* f. sp. *phaseoli infection*. Front. Genet..

[27] Ullah I., Magdy M., Wang L., Liu M., Li X. (2019). Genome-wide identification and evolutionary analysis of TGA transcription factors in soybean. Sci. Rep..

[28] Ji Q., Zhang L., Wang Y., Wang J. (2009). Genome-wide analysis of basic leucine zipper transcription factor families in *Arabidopsis thaliana*, *Oryza sativa* and *Populus trichocarpa*. J. Shanghai Univ. (Engl. Ed.).

[29] Thurow C., Schiermeyer A., Krawczyk S., Butterbrodt T., Nickolov K., Gatz C. (2005). Tobacco bZIP transcription factor TGA2.2 and related factor TGA2.1 have distinct roles in plant defense responses and plant development. Plant J..

[30] Qi P., Huang M., Hu X., Zhang Y., Wang Y., Li P., Chen S., Zhang D., Cao S., Zhu W. (2022). A Ralstonia solanacearum effector targets TGA transcription factors to subvert salicylic acid signaling. Plant Cell.

[31] Gaudinier A., Rodriguez-Medina J., Zhang L., Olson A., Liseron-Monfils C., Bågman A.M., Foret J., Abbitt S., Tang M., Li B. (2018). Transcriptional regulation of nitrogen-associated metabolism and growth. Nature.

[32] Chen C., Chen H., Zhang Y., Thomas H.R., Frank M.H., He Y., Xia R. (2020). TBtools: An integrative toolkit developed for interactive analyses of big biological data. Mol. Plant.

[33] Tamura K., Stecher G., Kumar S. (2021). MEGA11: Molecular evolutionary genetics analysis version 11. Mol. Biol. Evol..

[34] Lescot M., Dehais p., Thijs G., Marchal K. (2002). PlantCARE, a database of plant cis-acting regulatory elements and a portal to tools for in silico analysis of promoter sequences. Nucleic Acids Res..

[35] Ramirez-Gonzalez R.H., Borrill P., Lang D., Harrington S.A., Brinton J., Venturini L., Davey M., Jacobs J., van Ex F., Pasha A. (2018). The transcriptional landscape of polyploid wheat. Science.

[36] Borrill P., Ramirez-Gonzalez R., Uauy C. (2016). expVIP: A Customizable RNA-seq Data Analysis and Visualization Platform. Plant Physiol..

[37] Liu Z., Xin M., Qin J., Peng H., Ni Z., Yao Y., Sun Q. (2015). Temporal transcriptome profiling reveals expression partitioning of homeologous genes contributing to heat and drought acclimation in wheat (*Triticum aestivum* L.). BMC Plant Biol..

[38] Li Q., Zheng Q., Shen W., Cram D., Fowler D.B., Wei Y., Zou J. (2015). Understanding the biochemical basis of temperature-induced lipid pathway adjustments in plants. Plant Cell.

[39] Enrico P., Tanzarella O.A., Paolacci A.R., Mario C. (2009). Identification and validation of reference genes for quantitative rt-pcr normalization in wheat. BMC Mol. Biol..

[40] Udvardi M.K., Czechowski T., Scheible W.R. (2008). Eleven golden rules of quantitative RT-PCR. Plant Cell.

[41] Liu P., Xu Z.S., Pan-Pan L., Hu D., Chen M., Li L.C., Ma Y.Z. (2013). A wheat PI4K gene whose product possesses threonine autophophorylation activity confers tolerance to drought and salt in Arabidopsis. J. Exp. Bot..

[42] Clough S.J., Bent A.F. (1998). Floral dip: A simplified method for Agrobacterium—Mediated transformation of *Arabidopsis thaliana*. Plant J..

[43] Schneider C., Rasband W., Eliceiri K. (2012). NIH Image to ImageJ: 25 years of image analysis. Nat. Methods.

