# Supplementary figures and images for: Functional Identification Reveals That TaTGA16-2D Promotes Drought and Heat Tolerance
Source: Plants (Basel). 2025 Jul 9;14(14):2125. doi: 10.3390/plants14142125 (PMC12300423; doi:10.3390/plants14142125)

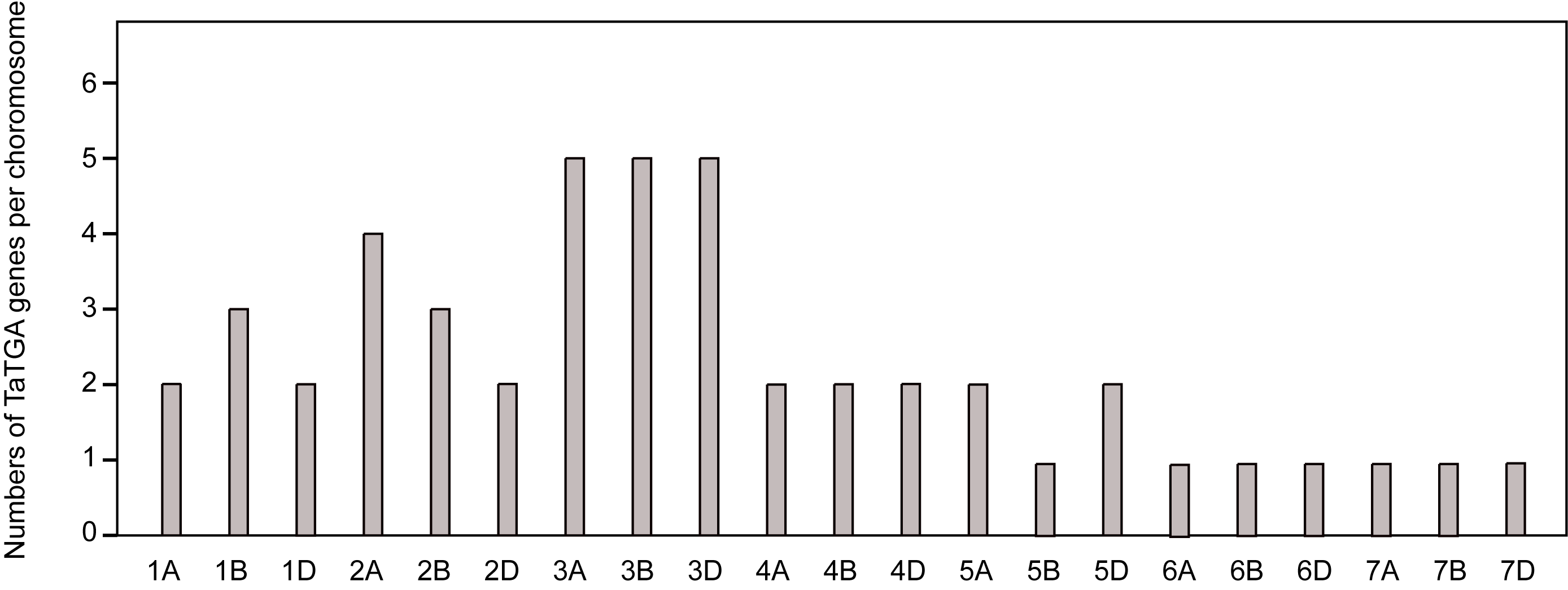

Supplement: Supplementary file 1 [file plants-14-02125-s001.zip › Supplementary Figure S1.tif]

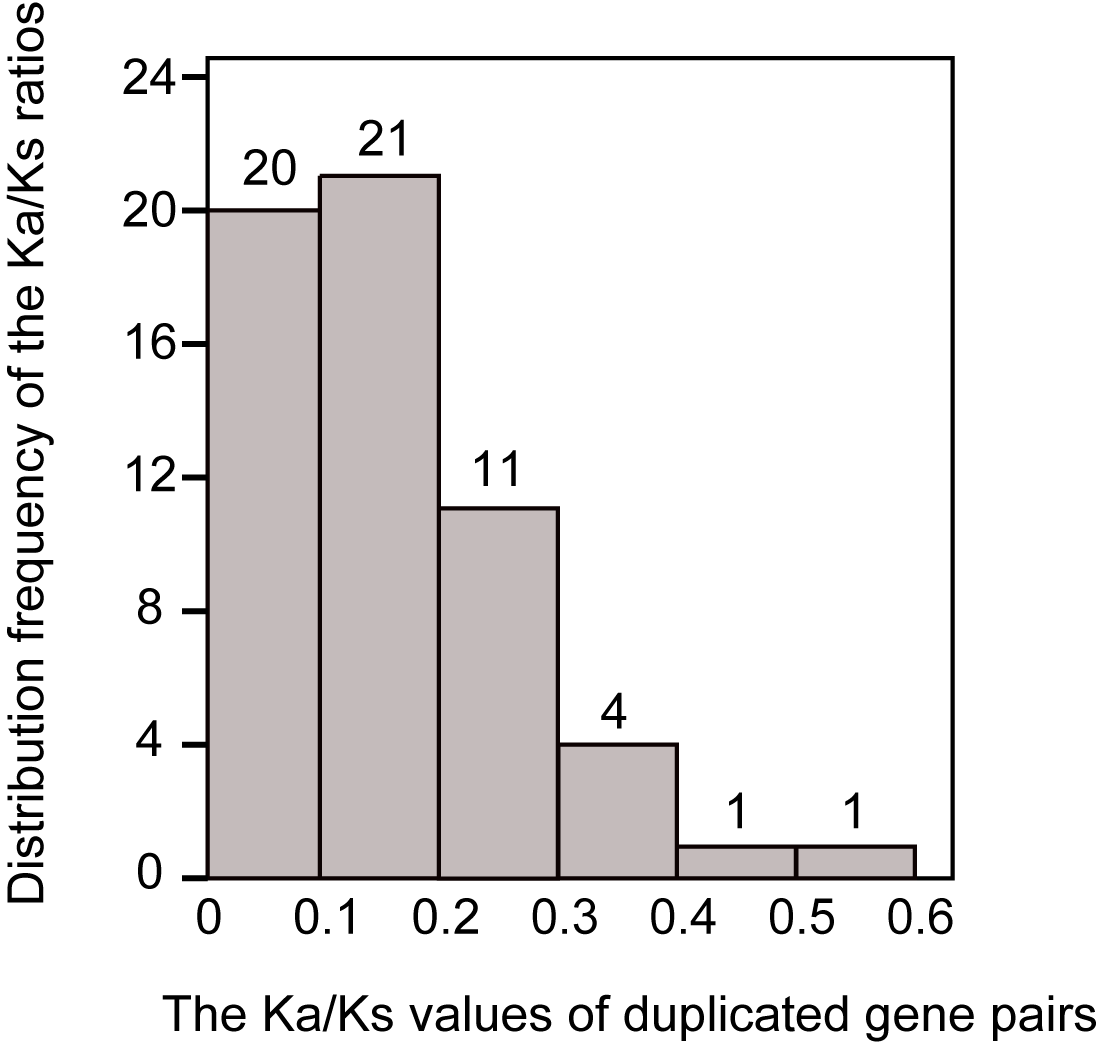

Supplement: Supplementary file 1 [file plants-14-02125-s001.zip › Supplementary Figure S2.tif]

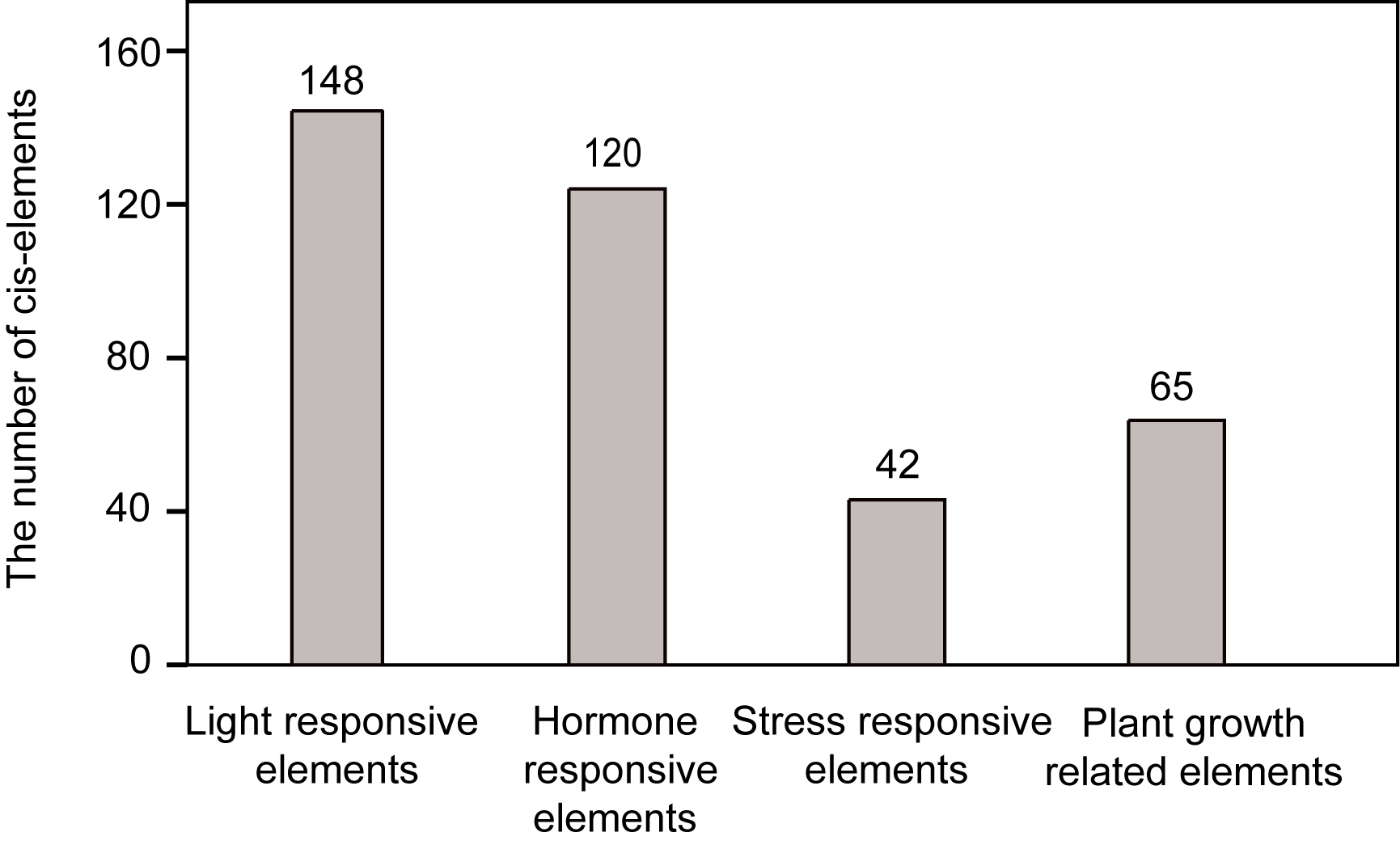

Supplement: Supplementary file 1 [file plants-14-02125-s001.zip › Supplementary Figure S3.tif]
